# The Importance of Synthetic Associations Will Only Be Resolved Empirically

**DOI:** 10.1371/journal.pbio.1001008

**Published:** 2011-01-18

**Authors:** David B. Goldstein

**Affiliations:** Center for Human Genome Variation, Duke University School of Medicine, Durham, North Carolina, United States of America

Before we had the tools to systematically interrogate variation throughout the human genome, there were two schools of thought in sometimes mortal combat. As Robert Shields reminds us in his editorial, some argued for the common-disease common-variant model (CD-CV), postulating an important role for common variants in common disease, while others argued that a great diversity of different rare variants were most likely the primary drivers of common diseases. The International HapMap Project, and the genome-wide association studies (GWAS) it enabled, were motivated in part by the CD-CV model.

Before GWAS, strong theoretical arguments were marshaled in support of either rare variants [1] or common variants [2], but few data were available to resolve the dispute. GWAS changed that by allowing us a (nearly) comprehensive evaluation of the role of common variation in human disease.

It is worth noting that GWAS have been serving their intended purpose remarkably well. It is generally agreed that GWAS successfully represent most of the common variation in the human genome. Moreover, the sample sizes that have now been analyzed for most common diseases would allow detection of most of the common variants that have even a modest impact on disease risk. For many common diseases, however, the cumulative impact of common variants implicated to date is modest, leading to the “missing heritability” question [3].

Another key observation has been the pathogenicity of copy number variants (CNVs). Here, we see variants that are rare by anyone's definition that have a dramatic impact on risk for many neuropsychiatric diseases [4]. Indeed, the effect sizes that have been associated with some of these rare risk factors are dramatically beyond what many would have previously considered realistic for such complex diseases. Together, these two observations, amongst others, led me to wonder whether we were interpreting the results of GWAS correctly.

In our first paper evaluating the properties of synthetic associations [5], we sought to address a very simple question: if a significant GWAS signal is observed, is it justified to infer that a common variant must be responsible for that signal? Before our paper, many researchers explicitly or implicitly assumed just that. Our paper investigated the properties of synthetic associations in the context of GWAS, asking whether the sample sizes and significance thresholds being considered were such that rare variants could reasonably be considered as candidates for creating some of the GWAS signals. We concluded that rare variants can easily create genome-wide significant associations credited to more common variants given the sample sizes being considered today, and it is therefore unjustified to assume that a GWAS signal must reflect the effect of a common variant. We also noted the practical implication that if an association is indeed synthetic, it is possible that the causal variant is much farther away from the discovery variant than would be possible if the cause is a common variant. Although we can show that rare variants could create synthetic associations credited to common variants, this does not allow us to determine the proportion of GWAS signals that are synthetic. In my view, this is a question that will only be answered empirically by tracking down the causal variants (almost certainly through sequencing).

Before addressing the specific comments by Anderson et al. and Wray et al. on our work, it is worth clarifying a few points that I consider to be essentially beyond reasonable argument:

1. **Most of the reported GWAS signals reflect true genetic associations**
  Some have recently suggested that many of the reported GWAS signals may be spurious in some sense. I have no sympathy for this perspective. I am wholly convinced that most of the broadly accepted GWAS signals reflect the presence of one or more real inherited genetic risks that are associated with the discovery variant. The suggestion that some of the GWAS signals are synthetic in no way calls into question the reality of GWAS signals.
2. **GWAS are a highly effective tool and were well worth doing**
  I consider both the HapMap project and the resulting GWAS phase to have provided crucial information about the genetic architecture of human diseases, and I consider it to have been the natural experiment to do.
3. **GWAS have made important discoveries**
  While the ultimate utility of many GWAS signals has been severely limited by the inability to move from a GWAS signal to causal variants in most cases, or by their poor diagnostic/prognostic value, there are many examples of GWAS discoveries that are of uncontested interest biologically and may have clinical significance.
  To these points of agreement we might add one more:
4. **Synthetic associations are plausible**
  Neither Anderson et al. nor Wray et al. contest the central fact that synthetic associations can work more or less as we described them. That is, given sample sizes considered today, variants substantially more rare than discovery variants can create signals of associations credited to common variants. Moreover, under synthetic associations, it is possible for the variants to be at some considerable physical distance from the associated variant.

These are then the points of agreement. Anderson et al. and Wray et al. both marshal a range of different arguments to conclude that synthetic associations as we describe them, while possible, are unlikely to be responsible for many of the GWAS signals that have been reported. They offer two primary arguments. Anderson et al. argue that variants that would drive synthetic associations should have been detected in linkage analyses for common diseases, while Wray et al. argue that the distribution of allele frequencies of variants implicated in GWAS is inconsistent with a model of synthetic associations that would favor association being credited to the lower frequency classes amongst the SNPs interrogated in GWAS. I address these key arguments in turn.

## Does the Absence of Widely-Accepted Linkage Evidence Rule Out Synthetic Association?

The argument is that variants with large enough effect sizes to drive synthetic associations should be detected in linkage studies, so if they are in the genome, why haven't we found them? There are two fundamental problems with this argument. The first is that our understanding of what linkage does and does not permit is more limited than suggested, in part because of how variable linkage results and interpretations have been. It is certainly true that relative risks above 4 should generate a strong linkage signal. But what has and has not been shown by linkage is more equivocal than implied. For example, it has been reported that most chromosomes have been implicated in linkage studies of bipolar disease and schizophrenia [6]. What fraction of these are real signal and what fraction are false positive? There is simply no way to know. CNVs are also instructive in this context. Even though some have very high relative risks and some are inherited, they were not “detected” in linkage studies (perhaps in part because of their nonspecifity).

The second problem with the argument is perhaps the stronger one. Even granting that the absence of widely accepted evidence in linkage should be taken as evidence of the absence of variants with relatively strong effects, this does not in fact rule out synthetic associations simply because of the extraordinarily large sample sizes used in many GWAS. We centered our analyses around a sample size of 2,000 cases and 2,000 controls. We showed that for such sample sizes it is very easy to get a synthetic association for a relative risk of 4, whether there was one or several rare variants in the range of frequencies considered (.005, .02). But even for this sample size and a relative risk of only 2, a small proportion of the gene genealogies still result in genome-wide significant associations credited to common variants. So what does this mean? It means that if there are many such rare variants of modest impact in the genome they will still create a few signals, even if most of them do not.

In fact, however, GWAS sample sizes for many traits have dramatically exceeded this size, meaning that weaker and weaker effects of rare variants will still easily create genome-wide significant synthetic associations. Indeed, if one simulates 25,000 cases versus 25,000 controls, rare variants with a relative risk of only 2 will *usually* create genome-wide significant synthetic associations (unpublished data). This observation is really just a testament to the singular power of detection of contemporary GWAS.

In short, even if we presume that all past linkage studies have been performed and interpreted correctly, these arguments make clear that it is easy to imagine a fair number of rare variants escaping linkage detection and yet driving synthetic associations, and this becomes increasingly likely as the sample size increases. Thus, we may well expect that synthetic associations make a relatively greater contribution to the signals observed in the more recent studies that have employed exceptionally large sample sizes, for example for lipids [7], body mass index [8], and height [9].

## Does the Allele Frequency Distribution of Associated Variants Rule Out Synthetic Associations?

Wray et al., on the other hand, argue that if most associations responsible for GWAS were synthetic, then the implicated common variants would be rarer than what has been observed. To arrive at this conclusion they simulate synthetic associations using the same approach we used, and sample only a subset of variants in order to match the allele frequency distribution of the variants on GWAS chips. They then show that under synthetic associations the mean frequency of the most associated genotyped SNPs is 0.13 for one rare causal variant and 0.3 for up to 18 causal variants. The authors argue (rightly) that this distribution is different from the distribution of allele frequencies of the discovery variants in GWAS, which tend to be the more common variants. But what may we conclude from this formally? If we assume that the simulated allele frequency distribution the authors use actually matches all the various GWAS chips used in various studies, and if we further assume that investigators have no biases in terms of what variants they choose to follow up in replication analyses, then we may conclude that the distribution of allele frequencies of discovery variants is inconsistent with a model in which synthetic associations are responsible for *all* GWAS signals. That is, we need to have at least *some* of the GWAS signals due to common variants in order to pull the overall distribution upward from what would be expected from an absolutely rare-only model for *all* associations. As far as I am aware, no one has ever articulated a rare-only model for *all* GWAS signals. The natural question then is: what proportion of the GWAS signals would have to be due to common variants to perturb the overall allele frequency distribution away from the expectation of a rare-only model? This has not been investigated, but it seems quite likely that such an investigation would leave open plenty of scope for synthetic associations to contribute to some, and perhaps many, signals, as we suggested [5]. Thus, of the hundreds of GWAS signals now listed on the National Human Genome Research Institute Web site, might synthetic associations be responsible for a substantial number of signals without pulling the entire allele frequency distribution out of the common range? It would certainly seem so.

In addition to these central arguments, both Anderson et al. and Wray et al. make a number of secondary arguments. Wray et al. also highlight the analyses of the International Schizophrenia Consortium (ISC), and suggest that we implied that a rare-only model is sufficient to explain their results. Addressing the inferences of the ISC is beyond the scope of this response, but it is worth clarifying that what I question is that the ISC analyses prove “a substantial polygenic component to the risk of schizophrenia involving thousands of common alleles of very small effect”, as they stated [10]. Under a model involving a contribution of synthetic associations, some of that signal credited to thousands of common alleles of tiny effect could be coming from rare variants of larger effect, and I cannot see how this could be ruled out.

The authors also note that many GWAS for height are near genes influencing skeletal growth. This, however, in no way points towards the cause of the associations being rare or common. We have shown that if the causal variant (or variants) is rare, then it is possible for the discovery variant to be at some distance removed from the causal variant [5]. Nevertheless, there is no reason to assume that when the causal variant is rare, the discovery variant should be preferentially far from the causal variant. Indeed, the expected association between any causal variant (rare or otherwise) and other variants decreases with distance. Simply because the causal *can* be farther away under synthetic associations does not mean it always is. Thus, the proximity of skeletal growth genes in the height GWAS data is perfectly consistent with either rare or common variants driving those signals.

Finally, Wray et al. point out that for the genetic models we simulated, if all associations for a given trait were synthetic, then this would impose a constraint on the total number of genomic regions that could be so mapped before the full genetic control of a trait were explained (because the genetic contribution of a region is greater than it appears if the source of the signal is synthetic). This constraint, however, still easily allows the possibility that many of the reported signals are synthetic and does not lend itself to any estimator of what proportion of signals for most traits might be synthetic. In their commentary, Anderson et al. highlight the fact that the WTCCC sequenced 16 GWAS regions (hundreds of kilobases to a Mb around significant SNPs) and found no rare causal variants. This claim is a little hard to assess, as the analysis has not been published so far as I am aware, and only reported at meetings beginning in 2009. What is interesting, however, is that these follow-up efforts not only found no rare causal variants, they also found no common causal variants. While rare variants may be easier to recognize than common ones, they also may be much farther away from the discovery variant and they may be present on only a small proportion of the chromosomes that carry the risk allele of the discovery common variant. For these reasons, failure to find either rare or common causal variants does not constitute strong evidence for or against synthetic associations.

Anderson et al. also note that many of the GWAS signals are common across populations, suggesting they are due to common variants. There is, however, no guarantee that synthetic associations would always be population specific. It is easy to imagine relatively rare variants sometimes being shared amongst different Eurasian populations, and sometimes not being shared. Moreover, even when the rare variants are population specific, it is possible that similar signals are sometimes created by different rare variants in different populations in the same genome regions, and only detailed characterization of the pattern of association in different populations could reveal that.

So where does all this leave us? More or less where we started. It is unjustified to assume that any given GWAS signal is due to a common variant, as was usually done before our work. Equally, it is unjustified to assume that any given GWAS signal must be synthetic in origin. In fact, the proportion of GWAS signals that are synthetic in origin depends on the genetic architecture of human traits, and this architecture remains largely unknown. What we do know is that common variants of large effect have not been observed for many of the common diseases studied by GWAS, and that even the common variants that have been associated cannot be simply assumed to reflect the effect of a common variant until that variant is actually identified and shown to be causal. Thus, an answer to the question of what is responsible for GWAS signals will only emerge as we track down the precise gene variants that influence human traits and then put them back into the context of GWAS signals to settle what may be, by then, simply an interesting historical footnote. On the basis of all current evidence, I certainly lean toward thinking that rarer variants are generally more important than common ones in common diseases, although this is ultimately an empirical question. Our work on synthetic associations was designed to show that they can happen, and since they can happen quite readily, I believe they must be responsible for some GWAS signals, and perhaps many. But we have no way of knowing whether it is a majority or minority. Time will tell.

Finally, it is worth reemphasizing that even for those GWAS signals that are synthetic, they still make a valuable contribution to work going forward. Despite its limitation in representing mostly common variation, there is one critically important feature of GWAS. It is a highly accurate and well understood experiment. A GWAS signal emerging from a properly conducted study means there is at least one causal variant somewhere relatively nearby. This cannot be said about sequence data, which currently can sometimes feel more like the Wild West than the laboratory. GWAS signals, be they synthetic in origin or not, give us a key foothold in the early days of interpretation of complete human-genome sequence data. Sequencing is and should be the future of discovery genetics, but as we charge into that future, I am glad we are armed with a few GWAS signals to aid in the interpretation.

## Abbreviations

CD-CV: common-disease common-variant model
CNV: copy number variant
GWAS: genome-wide association studies
ISC: International Schizophrenia Consortium
